# Hsa_circ_0043532 contributes to PCOS through upregulation of CYP19A1 by acting as a ceRNA for hsa-miR-1270

**DOI:** 10.1186/s13048-024-01474-5

**Published:** 2024-07-22

**Authors:** Huimin Zhang, Jianye Fang, Yingxue Liu, Wenqian Zhu, Yangying Xu, Yu Zhang, Wei Shen, Duan Li, Cuifang Hao

**Affiliations:** 1 Centre for Reproductive Medicine, Women and Children’s Hospital, Qingdao University, Qingdao, China; 2 Branch of Shandong Provincial Clinical Research Center for Reproductive Health, Qingdao, China; 3 College of Medicine, Qingdao University, Qingdao, China; 4 The Second Hospital of Shandong University, Jinan, China; 5 College of Life Science and Technology, Qingdao Agricultural University, Qingdao, China

**Keywords:** circ_0043532, miR-1270, CYP19A1, Estradiol, PCOS

## Abstract

**Background:**

Polycystic ovarian syndrome (PCOS) accounts for about 75% of anovulatory infertility. The cause of PCOS is not clear. CircRNAs acting as miRNA sponges mediate the post-transcriptional regulation of multiple genes. CYP19A1 is a limiting enzyme in the ovarian steroidogenesis pathway. However, the mechanism of circRNAs regulating granulosa cell (GC) estradiol secretion in PCOS remains to be elucidated.

**Methods:**

Bioinformatics was used to predict the potential target miRNAs of circ_0043532 and target genes of miR-1270. Target miRNAs and mRNA expression were verified by qRT-PCR in GCs from 45 women with PCOS and 65 non-PCOS. Western blot, ELISA and dual-luciferase reporter assays were applied to confirm the substrate of miR-1270.

**Results:**

Circ_0043532 and CYP19A1 were significant up-regulation in GCs from patients with PCOS. The predicted target miRNAs of circ_0053432, miR-1270, miR-576-5p, miR-421 and miR-142-5p, were notably decreased in GCs from patients with PCOS. Mechanistic experiments showed that circ_0043532 specifically binds to miR-1270. MiR-1270 was negatively regulated by circ_0043532. Concomitantly, miR-1270 inhibited CYP19A1 expression and estradiol production, which could be reversed by circ_0043532 over-expression.

**Conclusion:**

We identified that circ_0043532/miR-1270/CYP19A1 axis contributes to the aberrant steroidogenesis of GCs from patients with PCOS. This study broadens the spectrum of pathogenic factors of PCOS, and circ_0043532 might be a potential therapeutic target for PCOS.

**Supplementary Information:**

The online version contains supplementary material available at 10.1186/s13048-024-01474-5.

## Introduction

Polycystic ovarian syndrome (PCOS) is the most common reproductive endocrine disorder, which accounts for about 75% of anovulatory infertility [1]. PCOS is a complex endocrine condition characterized by hyperandrogenism, menstrual irregularity and polycystic ovarian morphology [2–4]. In addition to inducing infertility, PCOS may increase the risk of developing complications such as obesity, high risks of endometrial cancer, metabolic syndrome, type II diabetes and cardiovascular disease, further threatening women's long-term healt [5–7]. Despite decades of extensive research, the pathogenesis of PCOS remains to be elucidated.

Increasing evidence suggests that impaired ovarian function, accompanied by aberrant steroidogenesis and inability to develop a dominant follicle, may contribute to the pathology of PCOS [8]. The famous two-cell, two-gonadotropin theory presumed that theca cells can secrete androgens depending on LH and aromatase enzyme in cumulus granulosa cells (GC) in response to FSH stimulation can convert androgens into estrogens [9, 10]. The granulosa cell is the vital modulator of follicle development through regulating the steroidogenesis, the growth of follicles within the follicular niche. The number of granulosa cells increases in each individual follicle throughout development. During the early stages of folliculogenesis, granulosa cells surround and provide growth regulators for oocytes [11, 12]. It has been reported that GCs play a key role not only in normal folliculogenesis, but also in the aberrant steroidogenesis and follicle development observed in PCOS [13, 14]. Although the functions of GCs have been extensively described, there are still many points of ambiguity regarding their modulation by different genes.

Circular RNAs (circRNAs), a new class of abundant endogenous noncoding RNAs, are characterized by closed annular structures lacking a canonical 5’ cap or 3’ poly A tail [15]. Increasing evidence suggests that circRNAs participate in various diseases and physiological processes by acting as competitive endogenous RNAs (ceRNAs) and interacting with proteins under certain circumstances. The ceRNA hypothesis posited that circRNAs act as miRNA sponges because they can bind miRNAs and inhibit miRNA regulatory function [16, 17]. Notably, differential expressed circRNAs have been identified in PCOS patients. Mechanistic studies have showed the regulatory role of circRNAs in the proliferation and viability of GCs from patients with PCOS [18, 19]. However, whether differentially expressed circRNAs contribute to the aberrant steroidogenesis of GCs from patients with PCOS is still poorly understood.

We previously identified that circ_0043532 was significantly upregulated in GCs from patients with PCOS [20]. In the present work, functional studies revealed its regulatory role in steroidogenesis of GCs. Mechanistically, circ_0043532 sponged miR-1270 to stabilise CYP19A1 mRNA, resulting in estradiol overproduction and follicular development arrest. Our study suggests that circ_0043532 is involved in aberrant steroidogenesis of GCs and provides a new epigenetic perspective on PCOS pathogenesis.

## Materials and methods

### Clinical samples

According to the Rotterdam criteria (2004), patients were diagnosed as PCOS with at least two of the following criteria; (1): polycystic ovaries, (2): anovulation and/or oligo-ovulation, (3): signs of clinical and/or biochemical hyperandrogenism with the exclusion of congenital adrenal hyperplasia, Cushing's syndrome and androgen-secreting tumors. During the period of April 2019 and December 2020, a total of forty-five patients (fourteen patients from Yantai Yuhuangding Hospital and thirty-one patients from Qingdao Women and Children’s Hospital) who satisfied the above criteria were classified as PCOS, and sixty-five women (eighteen patients from Yantai Yuhuangding Hospital and forty-seven patients from Qingdao Women and Children’s Hospital) were recruited as the non-PCOS group who sought assisted reproductive techniques primarily due to male factors or tubal problems. The participants were treated with in vitro fertilization (IVF) or intracytoplasmic sperm injection (ICSI). This study was approved by the Ethics Committee of the Affiliated Hospital of Qingdao University (Qingdao Women and Children’s Hospital and Yantai Yuhuangding Hospital). We obtained written informed consent from all participants. The clinical characteristics of all patients are exhibited in Table 1 and Table 2.

**Table 1:** Comparison of clinical characteristics and endocrine parameters in PCOS and non-PCOS patients for mRNA analysis

Basic parameters	PCOS (*n* = 31)	non-PCOS (*n* = 47)	*p*-values
Age (years)	30.0 ± 3.0	30.3 ± 2.5	NS
Infertility duration(yr)	2.7 ± 1.3	3.0 ± 1.8	NS
BMI (kg/m^2^)	24.8 ± 3.9	22.5 ± 3.2	< 0.001
FSH (mIU/ml)	7.88 ± 2.86	8.71 ± 2.95	NS
LH (mIU/ml)	6.68 ± 5.60	4.73 ± 2.72	NS
LH/FSH	1.07 ± 1.12	0.64 ± 0.42	NS
PRL (ng/ml)	18.03 ± 10.93	20.50 ± 9.36	NS
P (ng/ml)	0.43 ± 0.39	0.45 ± 0.35	NS
AMH (ng/ml)	6.39 ± 3.51	3.56 ± 1.98	< 0.0001
E_2_ (pg/ml)	39.79 ± 7.48	33.50 ± 11.68	NS
T (ng/ml)	1.22 ± 0.38	0.73 ± 0.36	< 0.0001
FBG (mmol/L)	5.01 ± 0.46	4.94 ± 0.40	NS
Antral follicle count	31.10 ± 12.66	16.36 ± 6.62	< 0.0001
oocytes obtained	17.59 ± 6.19	14.66 ± 7.12	< 0.05
Embryo rate^a^	0.39 ± 0.19	0.43 ± 0.14	NS

Data are presented as the mean ± SD, *p* < 0.05 is considered to be statistically significant

*BMI* body mass inde, *FSH* follicle-stimulating hormone, *LH* luteinizing hormone, *PRL* prolactin, *P* Progesterone, *AMH* anti-Müllerian hormone, *E*_*2*_ estradiol, *T* testosterone, *FBG* Fasting blood glucose

^a^Embryo rate = number of embryos/oocytes obtained

**Table 2:** Comparison of clinical characteristics and endocrine parameters in PCOS and non-PCOS patients for miRNA analysis

Basic parameters	PCOS (*n* = 14)	non-PCOS (*n* = 18)	*p*-values
Age (years)	31.4 ± 1.5	31.4 ± 1.9	NS
Infertility duration(yr)	3.8 ± 2.1	3.3 ± 1.9	NS
BMI (kg/m^2^)	25.7 ± 3.3	23.1 ± 3.7	< 0.05
FSH (mIU/ml)	6.01 ± 0.90	6.66 ± 1.22	NS
LH (mIU/ml)	11.66 ± 4.61	5.19 ± 2.25	< 0.0001
LH/FSH	1.93 ± 0.70	0.77 ± 0.26	< 0.0001
PRL (ng/ml)	14.78 ± 5.91	18.61 ± 6.37	< 0.05
P (ng/ml)	0.29 ± 0.13	0.44 ± 0.18	NS
AMH (ng/ml)	11.64 ± 6.43	4.54 ± 2.95	< 0.0001
E_2_ (pg/ml)	48.65 ± 26.65	42.76 ± 20.91	NS
T (ng/ml)	0.59 ± 0.11	0.29 ± 0.09	< 0.0001
FBG (mmol/L)	5.03 ± 0.40	4.73 ± 0.35	NS
Antral follicle count	34.33 ± 13.72	17.56 ± 6.65	< 0.0001
oocytes obtained	16.78 ± 6.78	13.66 ± 6.44	< 0.0001
Embryo rate^a^	0.47 ± 0.23	0.47 ± 0.19	NS

Data are presented as the mean ± SD, *p* < 0.05 is considered to be statistically significant

*BMI* body mass inde, *FSH* follicle-stimulating hormone, *LH* luteinizing hormone, *PRL* prolactin, *P* Progesterone, *AMH* anti-Müllerian hormone, *E*_*2*_ estradiol, *T* testosterone, *FBG* Fasting blood glucose

^a^Embryo rate = number of embryos/oocytes obtained

### Cumulus granulosa cell isolation

The follicles were aspirated by transvaginal puncture under ultrasound echo-guidance 36 h after hCG injected, and then the cumulus-oocyte complex (COC) was obtained. Cumulus granulosa cells surrounding a single oocyte were discreetly isolated by using a sharp needle. After rinsing in phosphate-buffered saline (HyClone, USA) three times, the GCs were stored at -80℃ until RNA extraction. The GCs isolated from the 14 PCOS and 18 control patients were used in miRNA qRT-PCR. The cumulus granulosa cells isolated from other patients (31 PCOS and 47 control patients) were used in mRNA qRT-PCR.

### Cell lines and cell culture

KGN cells (RCB1154, RIKEN, Japan) were cultured in DMEM/F-12 medium (SparkJade Science, China) supplemented with 10% (v/v) fetal bovine serum (FBS; PAN Biotech, Germany) and 1% penicillin-streptomycin solution (SparkJade Science, China). IOSE80 cells (BNCC340318) and COV434 cells (BNCC338036, BNCC, China) were cultured in DMEM (SparkJade Science, China). 293 T cells were kindly donated by Professor Shen Wei from Qingdao Agricultural University. Cells were cultured in a humidified atmosphere with 5% CO_2_ at 37℃.

### Target prediction and bioinformatics analysis

The targeted miRNA of circ_0043532 was predicted by Circinteractome (https://circinteractome.nia.nih.gov/mirna_target_sites.htmL) and starBase (http://starbase.sysu.edu.cn/index.php). And the targeted mRNA of miRNAs were predicted by miRWalk 2.0 (http://zmf.umm.uni-heidelberg.de/apps/zmf/mirwalk2/) which included miRNA-mRNA interaction information produced by several established miRNA prediction programs (i.e., RNA22 (https://cm.jefferson.edu/rna22/Interactive/), miRWalk, TargetScan (http://www.targetscan.org/), miRanda (http://www.microrna.org/microrna/home.do), RNAhybrid (https://bibiserv.cebitec.uni-bielefeld.de/rnahybr)) on 3' UTRs of all known human genes (*p* ≤ value 0.05) [21].

### Cell transfection

The mimics, inhibitors and the negative controls (NCs) were designed and synthesized by Genepharma (Shanghai, China). Circ_0043532 sequence was cloned and inserted into vector pEX-3 (GenePharma, Shanghai, China) to over-express circ_0043532. We seeded about 5 × 10^4^ KGN cells/well in a 6-well plate with DMEM/F12 added with 5% FBS. The KGN cells reached 70%-90% confluence after 24 h, then starved for 12 h with only DMEM/F12. And then the miRNAs, inhibitors and NCs were transfected into the KGN cells by Lipofectamine 2000 reagent (Invitrogen, USA) based on the manufacturer’s protocols. The Sequence of mimics, inhibitor and NC were listed in Table S1. Next, the transfected cells were incubated for 24 h until the transfection efficiency was determined by RT-qPCR.

### Estradiol assays

The transfected-KGN cells were cultured in DMEM/F-12 supplemented with 5% FBS, plus androstenedione (100 nM) as a substrate. After cultivating for 48 h, cell incubation medium (1 × 10^5^ cells) was collected and centrifuged, the supernatant was extracted. The secretion of estradiol was determined by enzyme-linked immunosorbent assays (ELISA) kit (KGE014, R&D Systems, USA). The ELISA was performed as the manufacturer’s protocol. Next, the absorbance was measured at 450 nm and 570 nm through a microplate reader (BioTek Instruments, USA). Each experiment was repeated three independent times. Four parameter logistic (4-PL) curve fit was used to calculate the results of the samples.

### RNA extraction and qRT-PCR

Total RNA was extracted from the cultured cell lines or granulosa cells using miRcute miRNA Isolation Kit (TIANGEN, China) following the manufacturer’s instructions. The quality and concentration of RNAs were determined by QuickDrop (Molecular Devices, USA). Only final products with absorbance ratios A260/A280 between 1.8 and 2.0 were recruited to be eligible for further experiments. Specific divergent primer spanning the back-splicing sites of circRNA was designed in Primer-BLAST (NCBI, USA). The SPARKscript II RT Plus Kit (SparkJade Science, China) and qPCR SYBR Green Pro Taq HS (Accurate Biotechnology, China) were used for mRNA and circRNA qRT-PCR. The miRcute Plus miRNA First-Strand cDNA Kit (TIANGEN, China) and miRcute Plus miRNA qPCR Kit (TIANGEN, China) were used to detecting the expression level of miRNAs. qRT-PCR was performed with QuantStudioTM5 (Applied Biosystems, USA). GAPDH was chosen as the internal reference for the detection of mRNA and circRNA expression levels, while miRNA levels were normalized by U6. Each sample in each group of qRT-PCRs was run in three times and the relative expression of each gene was calculated by 2^−ΔΔCT^ method. All primers used in this study were compounded by TSINGKE Biological Technology (Beijing, China). The sequences of paired primer were listed in Table S2.

### Dual-luciferase reporter assay

The wild type circ_0043532 containing the predicated target sites for predicted miRNAs (miR-576-5p, miR-1270, miR-142-5p) (WT-circ_0043532) and the mutant sequences (MUT-circ_0043532) were inserted into pmirGLO luciferase reporter vectors (GenePharma, Shanghai, China), respectively. The 3’-untranslated region (3’UTR) of *CYP19A1* containing the predicted binding site for predicted miRNA (miR-1270) (*CYP19A1*-3’UTR-WT) and the mutant sequences (*CYP19A1*-3’UTR-MUT) were also inserted into pmirGLO luciferase reporter vectors (GenePharma, Shanghai, China), respectively. 293 T cells were seeded in 48-well plates and cultured overnight then co-transfected with a luciferase reporter vector and miRNA mimics (GenePharma, Shanghai, China) by using Lipo2000 reagent (Invitrogen, Carlsbad, USA). After 48 h incubation at 37℃ with 5% of CO_2_, luciferase activity was detected using the Dual-luciferase Reporter Assay Kit (Vazyme, Nanjing, China) following the manufacturer’s protocol. The results were expressed as relative luciferase activity (Firefly Luciferase/ Renilla Luciferase).

### Western blotting

After transfection for 48 h, KGN cells were harvested and lysed on ice with RIPA lysis buffer (Beyotime, China) complemented with Phenylmethanesulfonyl fluoride (PMSF, Beyotime, China). Each protein sample was detached using 8% SDS-PAGE gel and then transferred onto a PVDF membrane. These membranes were blocked in 5% BSA (Solarbio, China) for 1.5 h at room temperature and incubated with the following primary antibodies specific against *CYP19A1* (ab18995, Abcam, Cambridge, MA, USA) or β-actin (Boster, China) all night at 4℃. Next these membranes were washed and incubated with secondary antibody (HRP-labeled Goat Anti-Rabbit IgG(H + L), A0208, Beyotime, China) at room temperature for 1.5 h. After washing, the bands were visualized by BeyoECL Plus (Beyotime, China). β-actin was as the endogenous control.

### Statistical analysis

All data in this study were presented as mean ± standard deviation (SD). GraphPad Prism version 8 was employed for data analysis. The Gaussian distribution of the continuous variables was tested by the Kolmogorov-Smirnov statistic. Student’s t-test was applied to analyze the differences, and a *p*-value < 0.05 was considered as statistically significant.

## Results

### Circ_0043532 promoted the estradiol secretion of GCs

**Fig. 1:**
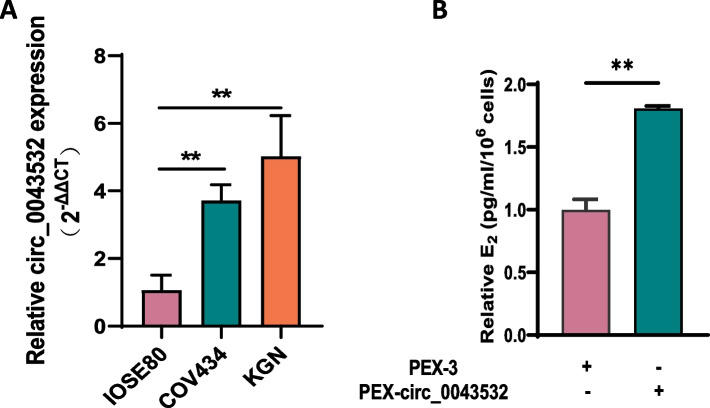
Circ_0043532 promoted the estradiol secretion of GCs. **A** qRT-PCR analysis of circ_0043532 in three different human ovarian granulose tumor cell lines. **B** Circ_0043532 potentiate estradiol production

We have previously identified that circ_0043532 was significantly upregulated in GCs from patients with PCOS. Before we conducted the functional experiments, the expression levels of circ_0043532 were measured in three different human ovarian GC cell lines. The results showed that circ_0043532 were highly expressed in KGN cells (Fig. 1A). Therefore, the KGN cell line was employed in the follow-up experiments.

To evaluated the impact of circ_0043532 on the steroidogenesis of GCs, we successfully over-expressed circ_0043532 in KGN cells (Figure S1A). Intriguingly, we found that the level of estradiol was significantly increased after circ_0043532 over-expression (Fig. 1B). These findings suggest that the aberrant steroidogenesis of GCs can be attributed to circ_0043532 over-expression.

### MiR-576-5p, miR-421, miR-142-5p and miR-1270 were the candidate target for circ_0043532

**Fig. 2:**
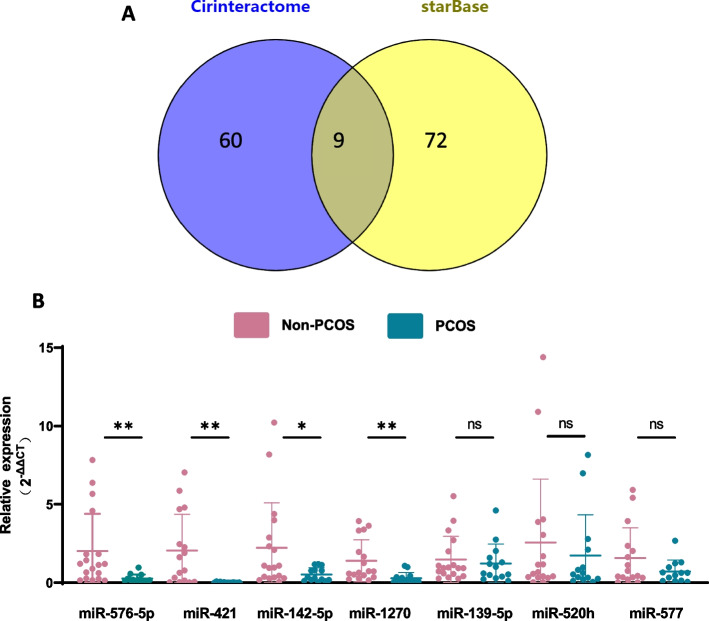
the candidate target miRNAs for circ_0043532. **A** The Venn diagram represents the mutual candidate target genes of circ_0043532 identified by Circinteractome and starBase. **B** Relative expression levels of candidate miRNAs were quantified by qRT-PCR between the PCOS and non-PCOS groups. The results are presented as the means ± SD. * indicates *p* < 0.05, ** indicates *p* < 0.01; ns indicates no significant difference

To identify the potential miRNA targets of circ_0043532, bioinformatics analysis tools of Circinteractome and starBase were utilized. Results showed that 9 miRNAs (miR-520 h, miR-142-5p, miR-1270, miR-577, miR-409-3p, miR-620, miR-576-5p, miR-139-5p, and miR-421) were predicted to be the potential targets of circ_0043532 (Fig. 2A). To evaluate the potential regulatory role of the target miRNAs, qRT-PCR was utilized to measure the expression levels of miRNAs in GCs from patients with PCOS. Specifically, 7 miRNAs (miR-520 h, miR-142-5p, miR-1270, miR-577, miR-576-5p, miR-139-5p, and miR-421) were selected for validation by qRT-PCR, and 2 miRNAs (miR-409-3p and miR-620) were excluded due to poor primer specificity. The qRT-PCR results showed that miR-576-5p, miR-421, miR-142-5p and miR-1270 were significantly decreased expression in GCs from PCOS patients (Fig. 2B).

### Circ_0043532 specifically binds to miR-1270

**Fig. 3:**
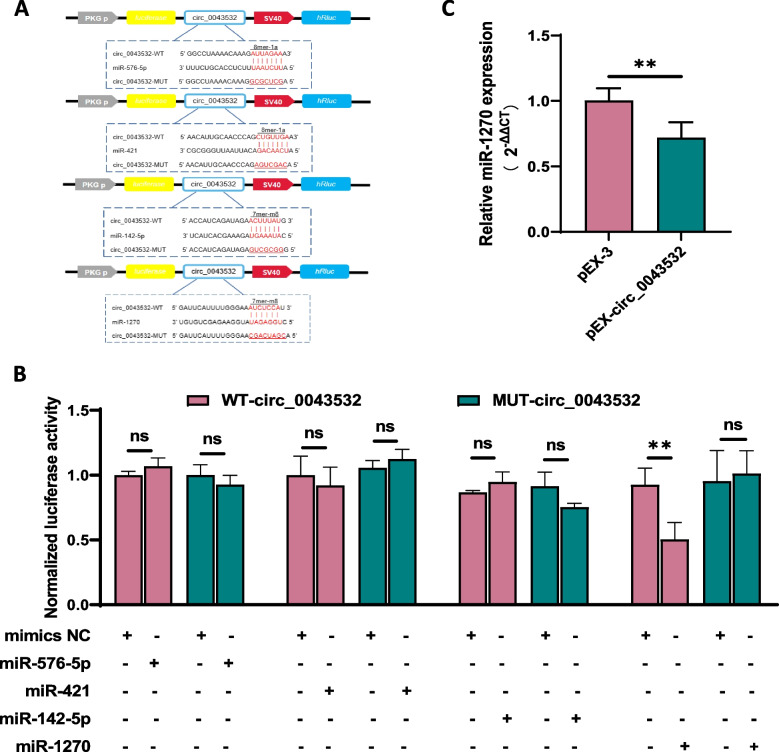
Circ_0043532 Specifically Binds to miR-1270. **A** Schematic showed the predicted binding sites between circ_0043532 and microRNAs in luciferase reporter, and wild-type or mutant binding sites were presented by red fonts. **B** Dual-luciferase reporter assay of WT-circ_0043532 or MUT-circ_0043532 after transfection with miR-576-5p, miR-421, miR-142-5p and miR-1270 mimics in 293 T cells. **C** Effects of pEX-circ_0043532 on the expressions of miR-1270 (*p* = 0.0088). The results are presented as the means ± SD. ** indicates *p* < 0.01; ns indicates no significant difference

To verify whether circ_0043532 sponges the four differential (decreased) expressed microRNAs (miR-576-5p, miR-421, miR-142-5p and miR-1270), we firstly predicted the potential target binding sites between circ_0043532 3’UTR and the miRNAs by using circinteractome, and the postulated binding sites between circ_0043532 and microRNAs were showed in Figure S1B. To further determine whether these four miRNAs bind to circ_0043532 3’UTR, we established luciferase reporters comprising either wild-type (WT) or mutant (MT) 3’UTR of circ_0043532 (Fig. 3A). Intriguingly, the luciferase intensity of WT circ_0043532 significantly decreased in miR-1270 mimics group while the intensity of miR-142-5p, miR-421 and miR-576-5p exhibited no such difference (Fig. 3B). Afterwards, we found the overexpression of circ_0043532 could effectively decrease the expression of miR-1270 (Fig. 3C). Collectively, our findings suggest that circ_0043532 specifically binds to miR-1270.

### Circ_0043532 promoted CYP19A1 expression by sponging miR-1270

**Fig. 4:**
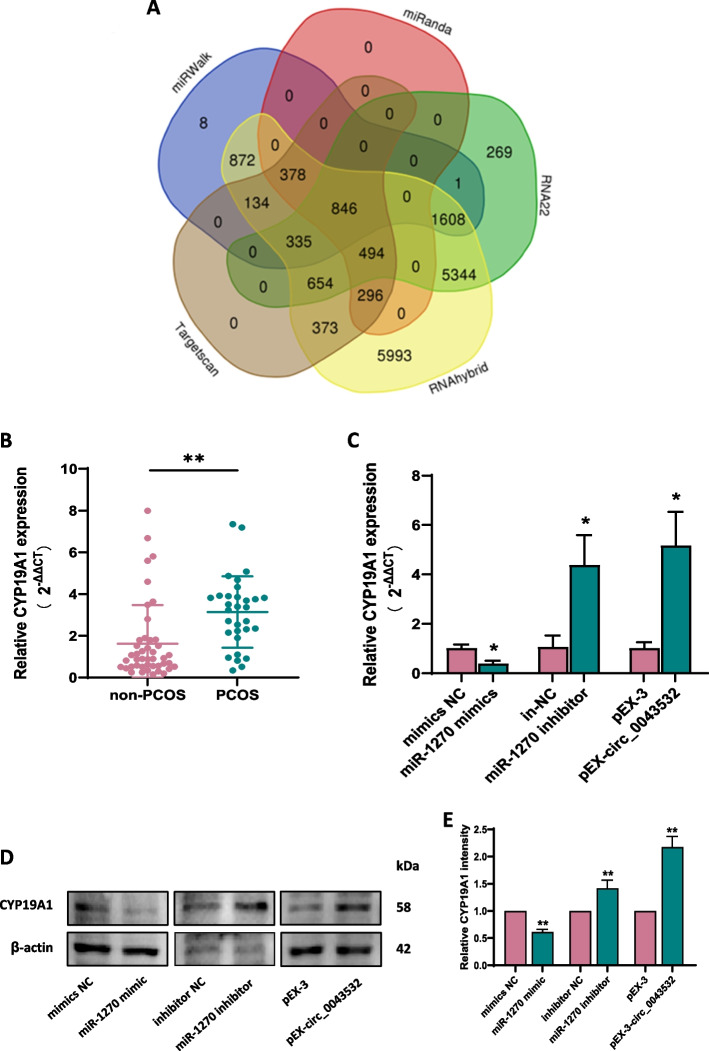
Circ_0043532 promoted CYP19A1 expression by sponging miR-1270. **A** The Venn diagram of potential target genes of miR-1270 predicted by miRWalk, RNA22, TargetScan, RNAhybrid and miRanda. **B** Relative expression levels of CYP19A1 quantified by qRT-PCR between the PCOS and non-PCOS groups. **C** Effects of miR-1270 mimics or inhibitor and pEX-circ_0043532 on the expressions of CYP19A1 were determined by RT-qPCR. **D**-**E** Western blot assay was performed to detect the expression of CYP19A1 after transfected with miR-1270 mimic or inhibitor and its NC as well as pEX-circ_0043532. The data are presented as the means ± SD. * indicates *p* < 0.05, ** indicates *p* < 0.01

To elucidate the mechanism through which circ_0043532 is involved in PCOS, five online tools (miRWalk, RNA22, TargetScan, RNAhybrid, and miRanda) were employed to predict potential targets of miR-1270, and 846 genes were listed in all five algorithms (Fig. 4A, Table S3). *CYP19A1*, among these genes, is a well-known limiting enzyme in the ovarian steroidogenesis pathway. Meanwhile, qRT-PCR analysis revealed a significant up-regulation of *CYP19A1* in GCs of derived from patients with PCOS (Fig. 4B). Therefore, we hypothesised that CYP19A1 is the potential target gene for circ_0043532 and miR-1270. Downstream experiments show that miR-1270 mimic or inhibitor effectively down-regulated or up-regulated the expression of *CYP19A1* in KGN cells, respectively (Fig. 4C). Furthermore, circ_0043532 significantly up-regulated the expression of CYP19A1, and down-regulated the expression of miR-1270, respectively (Fig. 3C, 4C). Consistently, western blot analysis uncovered that the expression of *CYP19A1* was down-regulated upon miR-1270 overexpression, and inhibition of miR-1270 or overexpression of circ_0043532 could enhance the protein levels of *CYP19A1* in KGN cells (Fig. 4D-E). The above data suggested that circ_0043532 promoted CYP19A1 expression through by sponging miR-1270.

### Circ_0043532/miR-1270/CYP19A1 axis contributes to the aberrant steroidogenesis of GCs from patients with PCOS

**Fig. 5:**
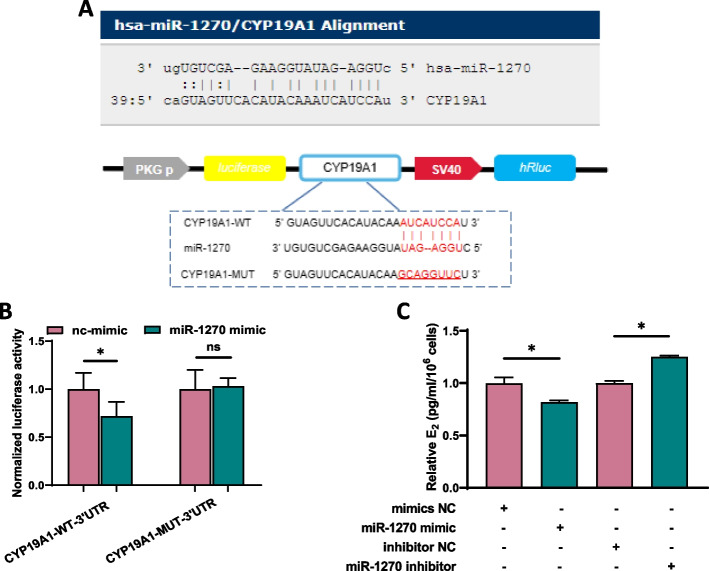
Circ_0043532 regulated the steroidogenesis via miR-1270/CYP19A1 axis in GCs. **A** Bioinformatics tool microrna.org revealed the predicted binding sites between CYP19A1 and miR-1270; CYP19A1 3'UTR sequences with wild-type or mutant miR-1270 binding sites were presented by red fonts in luciferase reporter. **B** Luciferase reporter assay demonstrated miR-1270 mimic significantly decreased the luciferase activity of CYP19A1-WT in 293 T cells. **C** The inhibitor of miR-1270 promoted E_2_ synthesis and the mimic of miR-1270 reduced estradiol production. The data are presented as the means ± SD. * indicates *p* < 0.05; ns indicates no significant difference

To reveal the binding capacity between miR-1270 and CYP19A1, microrna.org was utilized to analyse the binding sites between miR-1270 and the CYP19A1 3’UTR regions. Then luciferase reporters comprising either WT or mutant 3’UTR of CYP19A1 were established (Fig. 5A). The results revealed that miR-1270 significantly reduced the luciferase intensities of *CYP19A1* 3’UTR WT reporter. In comparison, co-transfection with MT reporter of *CYP19A1* 3’UTR and mimics of miR-1270 did not show a significant change in luciferase intensity (Fig. 5B). In addition, miR-1270 could markedly reduce the estradiol production whereas miR-1270 inhibitor could promote the estradiol synthesis, respectively (Fig. 5C). Collectively, our data demonstrated that circ_0043532 regulates the steroidogenesis via the miR-1270/CYP19A1 axis in GCs.

## Discussion

**Fig. 6:**
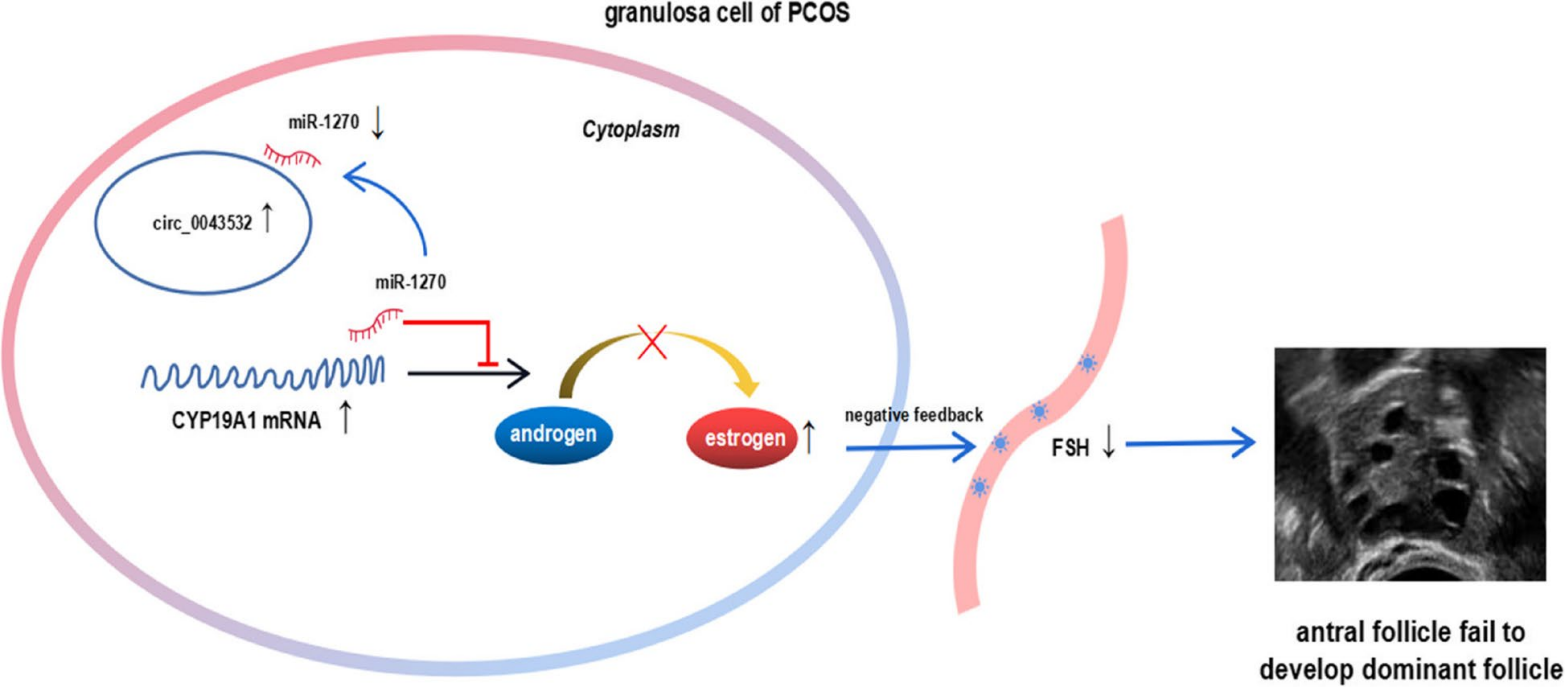
Circ_0043532/miR-1270/CYP19A1 axis contributes to the aberrant steroidogenesis of GCs from patients with PCOS. Image showing the underlying mechanism of how circ_0043532 is involved in the progression of ovarian steroidogenesis. Circ_0043532 acts as a ceRNA for miR-1270 to up-regulate CYP19A1, and miR-1270 down-expression further promotes the synthesis of estrogen

In the present study, we have demonstrated that overexpression of circ_0043532 in GCs promotes E_2_ synthesis, therefore further weakening the negative feedback effect of E_2_ on FSH secretion and resulting in arrested folliclar development and PCOS (Fig. 6). Mechanistically, circ_0043532 enhances *CYP19A1* expression by acting as a ceRNA for miR-1270, which would provide a novel therapeutic targets and strategies for PCOS. As the most conspicuous phenotypes of PCOS are aberrant steroidogenesis and abnormal follicular development, we believed that our findings provide a novel epigenetic perspective on the pathogenesis of PCOS.

Considerable evidence has verified that circRNAs regulate mRNAs expression through sponging miRNAs [22–24]. A recent study revealed that circ_0043533/miR-1179, circ_0030018/miR-136, circ_FURIN/miR-423-5p, circ-FURIN/miR-195-5p, circ_0043532/miR-182, circ_RANBP9/miR-136-5p, circRHBG/miR-515-5p, circMTO1/miR-320b, circASPH/miR-375, circPSMC3/miR-296-3p, circLDLR/miR-1294, circPUM1/miR-760, and hsa_circ_0118530/miR-136 as molecular axes contributing to the pathogenesis of PCOS [25]. For example, circ_0043532 promoted cells cell proliferation and cell cycle and suppressed cell apoptosis process by sponging miR-182, and the inhibition of miR-182 rescued the impacts of circ_0043532 interference on cell progression by targeting SGK3 [26]. However, our current study primarily focuses on elucidating the ceRNA mechanism of circ_0043532 in steroidogenesis in the current study. Firstly, Before the functional experiments were conducted, we found that the level of estradiol was significantly increased after circ_0043532 over-expression. We speculate that circ_0043532 may be involved in the steroidogenesis of GCs. Expression levels of potential target miRNAs of circ_0043532 were detected in GCs from patients with PCOS or without PCOS, of which miR-576-5p, miR-421, miR-142-5p and miR-1270 were notably decreased. In addition, we identified that circ_0043532 specifically binds to miR-1270, through which regulates the E_2_ synthesis. Numerous researches in recent years have discovered that miR-1270 serves as downstream target of circRNAs and therefore plays an indispensable role in various diseases. It was recently reported that hsa_circ_103809 accelerates hepatocellular carcinoma progression through miR-1270/*PLAG2* axis [27]. Circ_0001247 induces cervical cancer by regulating *ZEB2* via sponging miR-1270 [28]. Thus, our results shed new light on how circRNAs regulate the process of steroidogenesis in GCs.

The transformation of androgen to estrogen is limited by CYP19A1 activity [9, 10]. In addition, previous studies have proved that steroidogenic enzymes, such as *CYP11A1*, *CYP19A1* and *3BHSD*, are increased in GCs from the PCOS mice, concomitant with the increase of E_2_ and P [29]. What’s more, the expression of P450scc, *3BHSD*, and *CYP19A1* is also higher in GCs of PCOS and other animal models [30, 31]. We identified that *CYP19A1*, the rate-limiting enzyme in estrogen biosynthesis, is the direct target gene of miR-1270, and the expression of *CYP19A1* was significantly higher in GCs from patients with PCOS. We discovered a negative pertinence of miR-1270 and *CYP19A1* expression in GCs, that is, *CYP19A1* was down-regulated with miR-1270 overexpression, which provided evidence for the regulatory role of miR-1270 on steroidogenesis of GCs.

Polycystic ovarian morphology derives from the excessive secretion of estrogen in GCs. Therefore, aromatase inhibitors, such as letrozole, are widely used in the clinic for the induction of PCOS ovulation [32]. Given that estrogen is the final production of the ovarian steroidogenesis pathway, so aromatase might act as a therapeutical target for PCOS. Here, we identified that circ_0043532 overexpression upregulated CYP19A1 by competitively binding with miR-1270, and the synthesis of E2 was promoted. Women with PCOS are known to have abnormal follicular development arrested at the stage of antral follicle [33, 34]. The potential action mechanism for letrozole treated PCOS for infertility may be that the inhibition of aromatase can restrain estrogen production and further weaken the estrogenic negative feedback effect to hypothalamic/pituitary axis. This study provides strong clinical evidence for the use of aromatase inhibitors to induce ovulation in PCOS.

## Conclusion

In summary, we have proposed a new epigenetic mechanism in the pathogenesis of PCOS in which circ_0043532 promotes estradiol secretion via the miR-1270/CYP19A1 axis in human GCs. This study broadens the spectrum of pathogenic factors of PCOS, and circ_0043532 might be a potential therapeutic target for PCOS.

### Supplementary Information

Supplementary Material 1: Figure S1A. Relative expression levels of circ_0043532 quantified by qRT-PCR.** B. **Bioinformatics tool Circinteractome revealed that miR-576-5p, miR-421, miR-142-5p and miR-1270 were the potential targets for circ_0043532. The data are presented as the means ±SD. ns indicates no significant difference.

Supplementary Material 2: Table S1. Sequences of mimics, inhibitor and NC.

Supplementary Material 3: Table S2. Sequences of paired primer for RT-qPCR.cc

Supplementary Material 4: Table S3. The 846 potential targets genes of miR-1270 in all five algorithms (miRWalk, RNA22, TargetScan, RNAhybrid, and miRanda).

## Data Availability

No datasets were generated or analysed during the current study.

## Abbreviations

PCOS: Polycystic ovarian syndrome
GC: Granulosa cell
circRNA: Circular RNAs
miRNA: MicroRNA
COC: Cumulus-oocyte complex
NCs: Negative controls
UTR: Untranslated region
WT: Wild-type
MT: Mutant
RT-qPCR: Real-time quantitative polymerase chain reaction
