# Supplementary figures and images for: Hsa_circ_0043532 contributes to PCOS through upregulation of CYP19A1 by acting as a ceRNA for hsa-miR-1270
Source: J Ovarian Res. 2024 Jul 22;17:151. doi: 10.1186/s13048-024-01474-5 (PMC11265019; doi:10.1186/s13048-024-01474-5)

## Slide 1
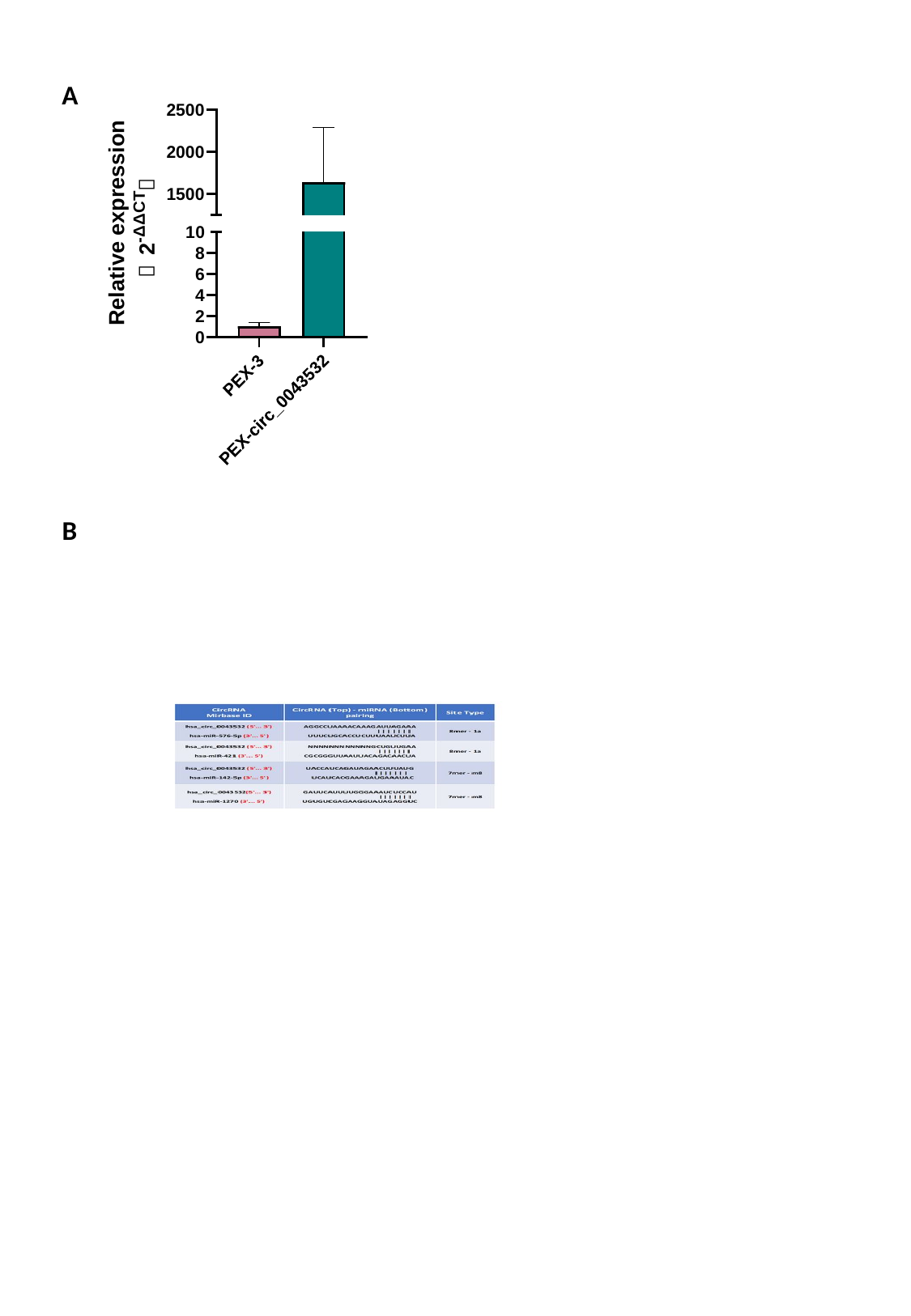

A
B

Supplement: Supplementary file 1 — Supplementary Material 1: Figure S1A. Relative expression levels of circ_0043532 quantified by qRT-PCR. B. Bioinformatics tool Circinteractome revealed that miR-576-5p, miR-421, miR-142-5p and miR-1270 were the potential targets for circ_0043532. The data are presented as the means ±SD. ns indicates no significant difference. [file 13048_2024_1474_MOESM1_ESM.pptx]
