# Supplementary material for: Hsa_circ_0043532 contributes to PCOS through upregulation of CYP19A1 by acting as a ceRNA for hsa-miR-1270
Source: J Ovarian Res. 2024 Jul 22;17:151. doi: 10.1186/s13048-024-01474-5 (PMC11265019; doi:10.1186/s13048-024-01474-5)
Supplement: Supplementary file 2 — Supplementary Material 2: Table S1. Sequences of mimics, inhibitor and NC. [file 13048_2024_1474_MOESM2_ESM.docx]

**Supplemental table** **Ⅰ**

Sequences of mimics, inhibitor and NC

| Supplement table Ⅰ\| miRNA-mimics、inhibitor and NC | |
| --- | --- |
| Gene | Sequence |
| mimics-NC | UUCUCCGAACGUGUCACGUTT |
|  | ACGUGACACGUUCGGAGAATT |
| miR-1270 mimic | CUGGAGAUAUGGAAGAGCUGUGU |
|  | ACAGCUCUUCCAUAUCUCCAGUU |
| miR-142-5p mimic | CAUAAAGUAGAAAGCACUACU |
|  | UAGUGCUUUCUACUUUAUGUU |
| miR-421 mimic | AUCAACAGACAUUAAUUGGGCGC |
|  | GCCCAAUUAAUGUCUGUUGAUUU |
| miR-576-5p mimic | AUUCUAAUUUCUCCACGUCUUU |
|  | AGACGUGGAGAAAUUAGAAUUU |
| miR-1270 inhibitor | ACACAGCUCUUCCAUAUCUCCAG |
| inhibitor-NC | CAGUACUUUUGUGUAGUACAA |
