# Supplementary material for: Hsa_circ_0043532 contributes to PCOS through upregulation of CYP19A1 by acting as a ceRNA for hsa-miR-1270
Source: J Ovarian Res. 2024 Jul 22;17:151. doi: 10.1186/s13048-024-01474-5 (PMC11265019; doi:10.1186/s13048-024-01474-5)
Supplement: Supplementary file 3 — Supplementary Material 3: Table S2. Sequences of paired primer for RT-qPCR.cc [file 13048_2024_1474_MOESM3_ESM.docx]

**Supplemental table** **Ⅱ**

Sequences of paired primer for RT-qPCR.

| Supplemental table Ⅱ\| Primers for RT-qPCR | | |
| --- | --- | --- |
| Gene | Primer | Sequence |
| GAPDH | Forward | GAGTCAACGGATTTGGTCGT |
|  | Reverse | AATGAAGGGGTCATTGATGG |
| circ_0043532 | Forward | ATTGCAACCCAGCTGTTGAAGC |
|  | Reverse | AGGCAAAACTTCAGCCATTTGT |
| CYP19A1 | Forward | GACTTTGCCACTGAGTTGATTT |
|  | Reverse | CGATCAGCATTTCCAATATGCA |
| U6 | Forward | CTCGCTTCGGCAGCACA |
|  | Reverse | AACGCTTCACGAATTTGCGT |
| miR-142-5p | GCCCTAGAAAGCACTACTAAA | |
| miR-576-5p | GGCTAATTTCTCCACGTCTTT | |
| miR-1270 | GGAGATATGGAAGAGCTGTGT | |
| miR-421 | ATCAACAGACATTAATTGGGCGC | |
| miR-520h | TCAAGTGCTTCCCTTTAGAGT | |
| miR-139-5p | TACAGTGCACGTGTCTCCAGT | |
| miR-577 | GCGCAATATTGGTACCTGAAA | |
| UnivR-miR | GCTGTCAACGATACGCTACGTAACG | |
