# Supplementary material for: Hsa_circ_0043532 contributes to PCOS through upregulation of CYP19A1 by acting as a ceRNA for hsa-miR-1270
Source: J Ovarian Res. 2024 Jul 22;17:151. doi: 10.1186/s13048-024-01474-5 (PMC11265019; doi:10.1186/s13048-024-01474-5)
Supplement: Supplementary file 4 — Supplementary Material 4: Table S3. The 846 potential targets genes of miR-1270 in all five algorithms (miRWalk, RNA22, TargetScan, RNAhybrid, and miRanda). [file 13048_2024_1474_MOESM4_ESM.docx]

**Supplemental table** **Ⅲ**

The 846 potential targets genes of miR-1270 in all five algorithms (miRWalk, RNA22, TargetScan, RNAhybrid, and miRanda).

| ZHX3 | MYO9B | CRNKL1 | EVC | ABCD4 | CTDSP2 |
| --- | --- | --- | --- | --- | --- |
| NUDT16L1 | SPTBN2 | KLHL31 | PGR | SOX6 | RAPGEF5 |
| SCAMP5 | RIOK3 | RAP2B | CSRNP3 | GLRA3 | MVB12B |
| FAM115A | CDC42SE2 | GPRC5B | KITLG | DDR2 | HAS1 |
| POT1 | ZFX | ADARB1 | PHC1 | ATP9A | SF3A1 |
| CHID1 | FAM129A | ARHGAP30 | CYP2U1 | MPP7 | SLC2A13 |
| STEAP2 | PIP4K2C | MGAT4A | GABRA4 | PDE11A | IRGQ |
| LONRF2 | AHR | SORBS2 | RIC3 | MTG1 | GPR158 |
| PHKG2 | DCBLD2 | PPP3R2 | RBM33 | C1orf227 | COMMD2 |
| RGS5 | UBL3 | TBC1D30 | RAB3D | MON2 | CTDSP1 |
| FXN | PBX1 | GALNT2 | FCGR3A | GNG2 | NLGN3 |
| RGS17 | DAZ2 | CERS6 | MTL5 | EPHA10 | CYBRD1 |
| S100A11 | LMTK2 | TNFSF12-TNFSF13 | COPS7B | C1orf95 | ZDHHC18 |
| HAUS2 | SOD2 | AGPAT1 | MAP3K3 | TCEANC2 | KCNC1 |
| SLC22A13 | MBTD1 | FBXL17 | ZNF585A | RAB22A | STAU1 |
| ELN | WDR36 | SLAMF8 | TCEB3 | CDH6 | KLHL4 |
| C1orf116 | MIB1 | RCL1 | CSF3R | BSN | GPC5 |
| PRR23C | SASH1 | ZNF705D | PPP3R1 | UBE3B | IQCE |
| KRAS | SIGLEC14 | LSM10 | C1orf21 | ZC3HAV1 | SDC4 |
| TMEM170B | FAT3 | WSCD2 | NEUROD4 | FAM189A1 | SMC5 |
| THRB | SPTBN1 | CDK17 | ABCF2 | ZBTB20 | IL17C |
| TMEM167A | ZBTB8B | PHEX | TM9SF3 | KIF3A | NMT1 |
| KRTAP12-2 | HOXA10 | SFMBT2 | RRP1B | ZNF195 | ZNRF2 |
| SAE1 | S100A7A | SPARC | OLA1 | CSRNP2 | SAR1B |
| ZNF589 | ADORA3 | TMBIM6 | SRD5A3 | IL17RA | ENTPD1 |
| MGAT5 | ATP13A3 | BDKRB2 | COPZ1 | TAF2 | SYPL2 |
| MAP3K2 | ALDH8A1 | RALA | ST8SIA3 | RAB3IL1 | RPL27A |
| CYP4A11 | GALNT6 | DCTD | KPNA4 | TFCP2L1 | STK4 |
| COQ9 | UBE2W | RBPMS | TFEC | KLHL6 | BTBD10 |
| HELZ | MPEG1 | ADCY2 | JAKMIP3 | NEURL1B | MKX |
| KIAA1737 | KRTAP12-1 | ZDHHC11 | DSC3 | CAPZA1 | USP31 |
| SLC35B4 | DAZ3 | GHR | TRIM2 | ZNF25 | STARD13 |
| CCNT1 | PHACTR2 | SH2D1B | FBXL7 | NACC1 | MKI67 |
| NR3C1 | CBLB | CDK6 | TTC39C | SULT2A1 | MEGF9 |
| CDK19 | PRDM15 | ZNFX1 | BCAT1 | DNER | CASK |
| ZSWIM4 | ELK4 | WFS1 | DUSP3 | RNASEH2C | CHD7 |
| PRR14L | CDC42SE1 | GATS | DPCR1 | SMARCA5 | SLC1A2 |
| SUFU | ZNF587 | SLC35A3 | HIPK2 | ASB7 | DLX1 |
| ANGPTL1 | FHOD3 | SOGA1 | RAX2 | UNC5C | CTSE |
| TSPYL6 | XYLT1 | RRN3 | HLTF | SSH2 | MRI1 |
| ADM2 | STX12 | KIAA1671 | EDA | FNDC5 | ZNF449 |
| RAG1 | CHRM1 | UBN2 | PCCB | VRK2 | WDR65 |
| MBNL3 | E2F2 | SLC6A12 | PER1 | SMARCAD1 | AIFM3 |
| GABPA | ZCCHC14 | CCDC149 | ACSS1 | TBX4 | APH1A |
| PRRC2B | APPBP2 | PALM2 | CLSTN2 | CBFA2T2 | MTR |
| TNNI1 | FLRT3 | PCYOX1L | DCAF10 | SLC7A1 | LYZ |
| USP2 | IL1RAP | TJP1 | PTPRT | CTSD | HHAT |
| ATP12A | ENPP1 | CYB561D1 | TMEM145 | ST8SIA2 | NLGN4Y |
| MKLN1 | APOBEC3A | OLR1 | CLK3 | BTBD9 | PTPN4 |
| LIN28A | WRB | NDEL1 | HDLBP | RNF122 | CHRNE |
| CNTN3 | ZNF155 | CACNB4 | CHTF8 | REG4 | ITIH6 |
| SPN | LMOD3 | GPD1 | TSPYL4 | PDE4C | CHST10 |
| RORC | UBFD1 | DEDD2 | PSAPL1 | C8orf33 | EPM2AIP1 |
| WT1 | GABRB1 | COPS2 | NCAPG2 | RGS7 | XRRA1 |
| CLVS1 | KRTAP8-1 | EIF2AK1 | OXNAD1 | C6orf106 | NCMAP |
| ARHGAP26 | ZBTB7C | VAT1 | MALL | GPR124 | GRID1 |
| XPO7 | RFX2 | SENP5 | ERBB4 | EIF4EBP2 | IRF2BP2 |
| SLC1A1 | SECISBP2L | DPH2 | COG5 | IQSEC1 | EPM2A |
| CBX6 | TMEM140 | LAMB3 | CTSB | C1orf198 | FAM114A2 |
| ATXN1 | FAM78B | NUDCD3 | RASSF8 | SOWAHA | TXNL4B |
| LDOC1L | GAB2 | DIS3 | FOXK1 | PML | CBFB |
| PRKCA | GGA2 | SCN5A | RNF169 | EPB41L4B | NFIA |
| CDON | MINPP1 | ARHGAP5 | DGCR14 | CACNB2 | CLCN3 |
| CACUL1 | PHC2 | ADO | GBP2 | MRC2 | ATM |
| RNF44 | NPTXR | PNKD | ADAM12 | DCAF5 | FNBP1L |
| SYNCRIP | SLC29A1 | RAB11FIP1 | PHF8 | ZNF396 | H2AFV |
| KCNA2 | HDAC5 | YEATS2 | REST | NOP2 | SPOPL |
| ST6GAL2 | PTAFR | GXYLT1 | STAC | ORC6 | SOX11 |
| PELI3 | CMKLR1 | AIF1L | ARID1B | KIF1B | PSMA5 |
| FZD4 | SESTD1 | CSNK2A1 | HEPACAM | ZNF445 | MEF2C |
| HTR6 | SHISA9 | DCP2 | LY6H | SPRY3 | NFATC2 |
| RGS8 | RAMP3 | PDE3A | SLC7A11 | BCL11B | RGS18 |
| SPHKAP | WIPF1 | RIMS4 | HSDL2 | CLTB | NXT2 |
| OGT | ZNF701 | PLA2G2F | HSPA13 | PLXNA2 | NFASC |
| TAOK1 | CD274 | GCNT1 | XK | WSCD1 | CCL22 |
| POU3F2 | PCSK9 | CEP104 | ADCY1 | PLXNA4 | PPARD |
| FBXW8 | RHOF | SHISA3 | CCDC141 | TBC1D5 | FAM105B |
| ZNF841 | OPRD1 | TOP2A | PURG | IL6ST | TMEM109 |
| ESRP2 | PLEKHO2 | ERC2 | SAMD5 | EPHB1 | ZBTB24 |
| ARL8B | VPS45 | KHNYN | SOS1 | PLAU | PTPN18 |
| PRKX | SERF1B | SIPA1L3 | AGPAT4 | TPM1 | DSG1 |
| BLOC1S6 | ZNF436 | GATAD2B | ZSCAN22 | DAZ4 | RPS6KA3 |
| MAN1A1 | NTRK2 | CLIC4 | SH3PXD2B | KRT4 | ZNF10 |
| DDAH1 | SV2B | SATB2 | STC1 | EFCAB14 | ZNF516 |
| DISP2 | CBLN3 | TGFBR2 | CIT | SHARPIN | VPS52 |
| DNMT3A | IBA57 | CERS3 | PIGK | MYOCD | ZNF862 |
| GALNT1 | AMOT | TMC7 | TSPAN11 | TC2N | NUP214 |
| CPEB2 | MLLT6 | ADAMTS1 | TBCA | GALK2 | GRIN2D |
| SNX16 | C16orf45 | CORO2A | MFN2 | MAPK10 | ALDH1L2 |
| RND2 | RNMT | POM121C | LRRC27 | ZNF2 | TMLHE |
| TMEM106A | PBOV1 | UTP23 | LASP1 | PTPN7 | ZNF335 |
| SMO | ZNF75A | ABHD2 | UBXN4 | RASSF5 | RPL32 |
| GLI2 | ATP6V1G2 | DGKE | S100PBP | CFLAR | RABGAP1L |
| LITAF | AFP | RIMS3 | KRT76 | LGSN | KIF5A |
| NUP43 | C18orf54 | LARP4B | REXO2 | SAMD4A | SNTB2 |
| DAAM2 | SP7 | F2RL2 | WDR92 | ANKH | POLR1B |
| NOS1 | PTGDR | RAP1GAP2 | B4GALT1 | PTGFR | SELE |
| SUGP2 | TUBG2 | PSORS1C2 | ADIPOR2 | APOBEC3G | ZBTB44 |
| PLXND1 | DTX4 | TNRC6B | SLC25A42 | GPR155 | NCOA5 |
| SCN3B | CNTD2 | TBC1D14 | TIA1 | MED29 | DICER1 |
| SORBS1 | GPR4 | JMY | FGF9 | ANKFY1 | LIMCH1 |
| AP3M2 | FRS2 | C9orf139 | LCOR | TUBB | SNX19 |
| MMP8 | MYO9A | PNMA2 | SH3BP2 | MYSM1 | NDST3 |
| KNSTRN | DSC2 | TRIM65 | GRHL2 | ABCC4 | VBP1 |
| SURF6 | OTUD7B | RABL3 | RASAL2 | TBC1D25 | ULK2 |
| PARP15 | ZMYND19 | IL17RD | TFAM | MARCHF5 | SAMD11 |
| SLC23A2 | PKD2 | ZNF704 | DNAAF3 | GGA3 | TRIM31 |
| CREB5 | ZFR2 | C7orf10 | PPP1R12B | SHISA6 | IGF2 |
| CALCR | TRPM8 | GOPC | APOBEC3B | YWHAB | CNOT6 |
| RNF4 | TBC1D15 | KLRF1 | TRIM25 | TYW5 | AGPAT3 |
| GRIN2A | IREB2 | ACSF3 | KCNIP1 | CYFIP2 | RORA |
| GNG7 | UBE2B | PPP2R5A | FGF5 | JHDM1D | POU2F3 |
| KIAA0226 | HDAC9 | PRLR | CGGBP1 | EMC1 | ITCH |
| ITPRIP | SOGA3 | ZCCHC24 | HBS1L | ULBP1 | EARS2 |
| NUFIP1 | APRT | SDPR | DNAL4 | FCRL5 | UROS |
| TMEM87A | RAB6B | TRIM46 | ZNF831 | SLC25A53 | RBBP5 |
| PDE1B | SRCIN1 | UBE2G1 | CYB5D1 | GOLGA1 | NDUFAF5 |
| RAPH1 | NOS1AP | SH3BGRL2 | POU2F1 | ACADSB | UNC45B |
| MAP7D2 | KIAA1009 | LRRC20 | GFI1B | PTGS1 | GALNT10 |
| XG | HABP2 | C1QTNF1 | PCGF5 | ANO5 | FAM8A1 |
| SELT | KLHDC8A | TGM2 | LAMC1 | KCNG3 | CBX7 |
| CCNY | FAM168A | FAM120C | ATP1B3 | C8orf46 | LCT |
| LRRFIP2 | ATP2B2 | ZNF175 | KMT2A | SLC9A3R2 | PARP9 |
| ZC3H7B | PPP1R3D | PLXNB2 | DAZ1 | SON | TBX18 |
| PDPR | ZNF470 | IFI27 | MASP1 | MCCC2 | C6 |
| PURB | NRAS | SLFN13 | OSBPL7 | SDK2 | DTNA |
| NUBPL | NEBL | ANKRD13A | SLC17A7 | FAM155B | IAPP |
| SLIT3 | AR | KRTAP4-4 | CLCN6 | KIAA1467 | SLC2A10 |
| ADNP | PAFAH2 | SEC63 | KMO | WBP1L | INHBC |
| CDS2 | RNF170 | DAZL | ANTXR1 | GJA5 | FAM212B |
| HLA-DQB2 | C11orf57 | CACNA1E | SLC26A9 | MEMO1 | CEACAM1 |
| TNFSF4 | MAPKAPK2 | RTBDN | RNFT2 | SLC4A8 | PKIA |
| SGCB | P4HB | FKTN | LPP | LYNX1 | TNFSF13 |
| HGS | ANKRD39 | SRRM4 | CCSER2 | PKLR | ST14 |
| OR7D2 | PROM2 | CTNNA3 | PHF21A | ZNRF3 | CRISPLD1 |
| HDAC2 | MS4A3 | SYNPO2 | ENY2 | CD4 | ZNF697 |
| SF1 | VTCN1 | SEC22C | SLCO1A2 | PEG10 | TAP2 |
| GPR12 | ZNF592 | HAS3 | XIRP1 | CCKAR | CHST11 |
| TNPO1 | WWC1 | FAM107A | GNE | ARNT | NFIB |
| CAPRIN1 | ANGPTL2 | PPM1A | SLC4A5 | WDR31 | IL7R |
| SERF1A | SET | GRIN3A | NBL1 | ATRX | FAM102B |
